# An overview of proactive monitoring and management of respiratory issues in ataxia-telangiectasia in a specialist and shared care pediatric clinic

**DOI:** 10.3389/fped.2024.1479620

**Published:** 2024-12-23

**Authors:** Jayesh Mahendra Bhatt, Andrew Bush

**Affiliations:** ^1^ Nottingham University Hospitals NHS Trust, Children's Respiratory Service, Nottingham, United Kingdom; ^2^ Paediatrics and Paediatric Respirology, National Heart and Lung Institute, Faculty of Medicine, Imperial College London, London, United Kingdom

**Keywords:** Ataxia-telangiectasia, lung disease, bronchiectasis, aspiration, interstitial lung disease

## Abstract

Ataxia-telangiectasia (A-T) is an ultrarare autosomal recessive disorder and occurs in all racial and ethnic backgrounds. Clinically, children and young people with A-T are affected by sinopulmonary infections, neurological deterioration with concomitant bulbar dysfunction, increased sensitivity to ionizing radiation, immunodeficiency, a decline in lung function, chronic liver disease, endocrine abnormalities, cutaneous and deep-organ granulomatosis, and early death. Pulmonary complications become more frequent in the second decade of life and are a leading cause of death in individuals with A-T. Oropharyngeal dysphagia is common, progressive, and a risk factor for frequent respiratory infections. Immunodeficiency is non-progressive in most patients with A-T. If severe infections occur, one should be aware of other possible causes, such as aspiration. We provide an overview of current best practice recommendations, which are based on combinations of extrapolation from other diseases and expert opinion. These include proactive surveillance, monitoring, and early management to improve lung health in this devastating multisystem disease.

## Introduction

Ataxia-telangiectasia (A-T) is an ultrarare autosomal recessive disorder caused by mutations in the Ataxia-telangiectasia mutated (*ATM*) gene, located on the long arm (q) of chromosome 11 (11q22–23). A-T affects between 1 in 40,000–300,000 live births worldwide (1) The Orphanet registry estimates the average prevalence of A-T at 1/100,000 children (2). The most reliable estimates for the number of people with A-T, in the UK at least, are 3 per million (3), and hence individual clinicians are likely to have limited experience in treating the disease. A-T occurs in patients of all racial and ethnic backgrounds and is associated with premature death in most people afflicted with the disease. The ATM protein is a 350 kDa serine/threonine protein kinase that is essential for double-stranded DNA repair following double-stranded DNA breaks (DSBs). ATM is also a critical constituent of the DNA damage response (DDR) pathway and is involved in detecting elevated oxidative stress and responding to DNA damage that occurs from the production of reactive oxygen and nitrogen species (RONS) by playing a critical role in the cellular antioxidant response to elevated oxidative stress and inflammation (4). In the absence of the ATM protein, the DDR response is compromised and cellular damage from oxidative stress may be amplified (5). DSBs can occur following exposure to ionizing radiation and this has implications for the use of diagnostic methods but, more importantly, for the use of therapeutic radiation in people with A-T. Double-stranded DNA breaks also occur during V(D)J-recombination, which is integral to the adaptive immune response. V(D) J-recombination is the process by which antigen receptors are assembled using distinct variable (V), diversity (D), and joining (J) gene segments as building blocks for T-cell receptor immunoglobulin chains (6). V(D)J-recombination is thus required for the expansion of the antibody and T-cell receptor repertoire response to foreign pathogens. Classically, V(D)J-recombination defects result in severe combined immune deficiency (SCID) with a lack of T and B lymphocytes (7). However, because there are functional redundancies in V(D)J-recombination, a total absence of ATM does not usually cause the severe immunodeficiency phenotype found in individuals with SCID. A positive result on newborn screening for SCID that is subsequently found not to be due to SCID should lead to additional testing and the diagnosis of A-T can be made after genetic studies are performed. This is because some infants with A-T have sufficiently low circulating copy numbers of T-cell receptor excision circles (TRECs) to trigger an abnormal report in the assay that detects SCID. In the UK, a handful of children are diagnosed annually with A-T after newborn screening.

The clinical and laboratory immunodeficiency markers in A-T varies between patients and can change during life. In approximately 70% of patients with classical A-T, ATM deficiency results in primary immune deficiency (PID) with highly variable features. Most patients have humoral and cellular immune defects comprising immunoglobulin-A (IgA) deficiency, immunoglobulin-G2 (IgG2) and immunoglobulin-G deficiency (IgG), and lymphopenia with low numbers of total and naïve CD4 T cells. Truncating mutations that result in a total absence of ATM kinase activity (null mutations) are present in severely affected patients, while at least one missense or splice site mutation is present in patients with milder phenotypes, resulting in the expression of ATM with some kinase activity (8, 9).

Between 10% and 60% of patients with A-T exhibit the hyper-immunoglobulin M (IgM) phenotype, with low IgG and IgA but with normal or elevated IgM (10–17). Patients with the hyper-IgM phenotype have worse overall survival, but the presence of IgG2 or IgA deficiency does not influence survival in A-T (18). Normal to high IgM levels are present as these individuals cannot switch from the production of IgM to other immunoglobulin classes due in part to the abnormal class switch recombination process (18).

Immunodeficiency is non-progressive in most patients with A-T. If severe infections occur, one should be aware of other possible causes, such as aspiration (8, 19–23).

Clinically, people with A-T are affected by sinopulmonary infections, neurological deterioration with concomitant bulbar dysfunction, increased sensitivity to ionizing radiation, immunodeficiency, a decline in lung function, chronic liver disease, endocrine abnormalities, cutaneous and deep-organ granulomatosis, and early death (17, 24–27). Pulmonary complications become more frequent in the second decade of life and are a leading cause of death in individuals with A-T. Oropharyngeal dysphagia is common, progressive, and a risk factor for frequent respiratory infections.

Given the myriad of system involvement in A-T and the rarity of the condition, delayed diagnosis is not uncommon (28) and clinicians should consider requesting an alpha-fetoprotein level test and confirming the genetic diagnosis when faced with a child with suggestive symptoms and signs (Box 1).

Box 1 **When to consider and confirm a diagnosis of A-T.**

1. Positive newborn screening test for inborn error of immunity which is not due to SCID.
2. Combination of lymphopenia and hypogammaglobulinemia.
3. Sibling with a confirmed diagnosis of A-T.
4. Investigating a toddler with ataxia, which is progressive, or “clumsiness,” which is atypical or more noticeable as compared to their peers.

Neurological manifestations contribute to respiratory disease (24, 25). A detailed description can be found elsewhere (24), but Table 1 [adapted from van Os et al. and Petley et al. (24, 29)] summarizes the key manifestations.

**Table 1:** Neurological manifestations of A-T.

	Classical A-T	Time frame of onset in classical A-T	A-T variant
Cerebellar ataxia, wide-based walking (gait ataxia), nystagmus, dysarthria, dysphagia, wobbling of the head and trunk (titubation), and shakiness when reaching (dysmetria)	Early and progressive	Generally presents at 12–18 months of age	Mild or absent
Extrapyramidal movement disorders (dystonia and tremor)	Later and less common	Between 4 and 6 years of age	Common feature
Oculomotor apraxia and nystagmus	Later and less common	Between 2 and 7 years of age	Common feature
Polyneuropathy and motor neuron disease	Later and less common	Second decade of life	
Cognitive impairment	Not a dominant feature		Not studied

The respiratory manifestations (Table 2) of A-T are:

(a) acute and chronic respiratory tract infection, with the risk of bronchiectasis, which is multifactorial. Risk factors include immune deficiency, aspiration due to uncoordinated swallowing, and impaired secretion clearance due to respiratory muscle dysfunction. Bronchiectasis can develop even before 3 years of age (30). Frequent sinopulmonary infections in A-T have been reported since early case reports were published (31), even before the underlying genetic basis of the disease was recognized, and is a very well-recognized component of the A-T syndrome. Pleural effusion is uncommon and should lead to a search for infective (32) and non-infective etiology including malignancy (33, 34);
(b) aspiration syndromes, related to uncoordinated swallowing and abnormal head posturing, can lead to/worsen bronchiectasis (see below);
(c) scoliosis, especially in adolescence;
(d) in adolescence, some people with A-T are wheelchair-bound and develop decreased physical conditioning with subsequent shallow breathing and reduction of tidal volume and inability to completely exhale further aggravating restrictive lung disease;
(e) (much more rarely) interstitial lung disease (ILD)/pulmonary fibrosis;
(f) restrictive and obstructive lung disease may develop after chemotherapy for malignancy.

**Table 2:** Respiratory manifestations of A-T.

Underlying problem	Respiratory consequences	Time frame of onset
Immune deficiency (due to underlying disease or secondary to treatment of a malignancy)	Recurrent upper and lower airway infections. Bronchiectasis with a “vicious cycle” of infection, inflammation, and secondary impairment of mucociliary clearance. Potential lower airway contamination from upper airway infection.	In preschool years, this may be difficult to distinguish from the recurrent infections seen in normal children Bronchiectasis: The youngest age at which bronchiectasis was diagnosed was *<*3 years. The oldest child reported with no bronchiectasis was 108.0 months.
Bulbar dysfunction	Recurrent lower airway infection and bronchiectasis. Inefficient cough due to failure of coordinated glottal closure.	Later in the first decade to early second decade.
Abnormal chest wall/scoliosis	Respiratory muscle dysfunction. Respiratory failure, initially nocturnal then daytime also.	Late teens
Inspiratory muscle dysfunction	Atelectasis Weak cough Respiratory failure, initially nocturnal then daytime also.	Later in the first decade to early second decade.
Expiratory muscle dysfunction	Weak cough	Later in the first decade to early second decade.
Unknown	Interstitial lung disease	Late teens
Malnutrition	Increased susceptibility to infection. Respiratory muscle weakness.	Later in the first decade to early second decade.
Physical deconditioning due to reduced activity and ataxia	Impaired secretion clearance Atelectasis.	Later in the first decade to early second decade.
Iatrogenic	Toxicity of anti-cancer chemotherapy, radiotherapy, and other treatments such as corticosteroids.	Later in the first decade to early second decade.

## Patterns of respiratory care

There are no large adequately powered randomized controlled trials of any respiratory treatments in A-T so all recommendations are based on combinations of extrapolation from other diseases and expert opinion (25).

We have updated the 2015 European Respiratory Society statement on multidisciplinary respiratory management of A-T (25) with additional material that has been published subsequently (Box 2).

Box 2 **New research and information related to A-T since 2015.**

• Two further reviews on multidisciplinary management of ataxia-telangiectasia.
• A systematic review of the natural history of A-T.
• Recognition of new presentations of lung disease in A-T, for example, chylothorax.
• Use of new modalities for respiratory monitoring, such as sniff nasal inspiratory pressure and peak cough flow.
• Update on immune responses and recommendations on immunization.
• New management strategies for pulmonary disease including nebulized saline, anesthetic challenges, and oxygen usage during critical care.

Given the complexity of the respiratory complications of A-T, it is strongly recommended that all children are cared for by a respiratory pediatrician in a local tertiary regional center (25). In suitable circumstances, a shared care system may be operated with a local district general hospital, similar to the cystic fibrosis (CF) model. There are similarities (progressive lung disease, respiratory microbiology, and multisystem involvement such as liver disease and diabetes) and differences [multiple contributors and phenotypes of lung disease (see Table 2), progressive neurological disease, risk of different types of cancers, and radiation susceptibility] between A-T and CF. As in CF, monitoring and treating respiratory infections (even in the era of highly effective modulatory therapy for CF), multisystem A-T-specific health issues, such as diabetes, liver disease, neurological disease, cancers, and psychosocial issues require multidisciplinary care with input from the relevant specialists. It is considered good practice that, as with CF (35, 36), there is an annual review undertaken so as to have proactive surveillance and early detection of any complication arising from A-T. In a shared care, non-specialist center, this should be undertaken by the responsible lead clinician with guidance from the specialist center. In addition, we recommend that these patients have a respiratory review at least every 3 months and more frequently if the child is unstable.

Best practice recommendations for infection control, including hand hygiene, contact precautions, regular microbiological surveillance, and segregation in clinical and social settings to minimize the acquisition of common pathogens, should be followed.

The components of the review should include (25) the following:

• Assessment by an experienced pediatrician.
• Assessment of airway clearance techniques by a physiotherapist with a special respiratory interest.
• Assessments by a speech and language therapist, and dietetic review to ensure optimal nutrition, are essential.
• Culture of sputum or cough swab including for *Pseudomonas aeruginosa*.
• Availability of pulmonary function tests for those children able to perform the technique. The underlying neurological involvement can make it difficult to obtain reliable reproducible dynamic lung function tests that require effort, active cooperation, and coordination. Specifically, children with A-T may struggle to attain full inflation (inspiratory muscle dysfunction) and full expiration (expiratory muscle dysfunction). People with A-T who have late glottis closure will produce forced vital capacity (FVC), slow vital capacity (SVC), and total lung capacity (TLC) results that are underestimated, and lead to the forced expiratory volume in 1 second (FEV1)/FVC and residual volume (RV)/TLC being overestimated, in addition to an overestimated VA. Inspiratory muscle weakness also leads to underestimates of TLC and mean inspiratory flow (VI)/tidal volume (VT). However, several researchers have demonstrated that pulmonary function testing with certain modifications can be performed reliably and be reproduced in patients with A-T and these tests may be used to track the rate of decline in lung function over time (20, 37–40). The reproducibility of standard spirometry testing is increased by stabilizing the patient's head, holding their cheeks while the patient performs the forced expiratory maneuver, optimizing the fit of the mouthpiece, and assessing for leaks in the system. Thus, the majority of adolescents with A-T are then able to perform spirometry testing with minimal modifications. Flow–volume curves do meet recommended American Thoracic Society (ATS)/European Respiratory Society (ERS) reproducibility criteria but often fail to meet other standard recommendations for forced spirometry and will often underestimate respiratory function. Impulse oscillometry and the forced oscillation technique require minimal patient cooperation and are especially useful in those who cannot perform effort-dependent exhalation (41, 42). These techniques can be used to assess small or large airway obstruction, bronchodilator responsiveness, or response to bronchoprovocation. Such investigations are increasingly being used in children in clinical settings (41) for asthma, bronchopulmonary dysplasia, CF, and more recently in ILD (43–45). As such testing is becoming much more available, there are standard technical recommendations (46). Impulse osciliometry (IOS) has the potential to become a useful modality of lung function in people with A-T. Multiple breath washout is also being increasingly used to detect lung disease in patients with inborn errors of immunity (IEIs), including A-T and is more sensitive than spirometry (47). Despite the technical issues, there are now physiological tests to track changes over time, even in A-T patients who cannot perform standard pulmonary function test (PFT). The use of tracking peak flow and force expiratory time, in addition to peak cough flow may also be of clinical adjunct in following expiratory weakness and fatigue.
• Stringent follow-up lung function monitoring should be adhered to in survivors of cancer. Elevated serum levels of IL-6 and IL-8 in people with A-T have also been associated with an increased risk of malignancies and worse lung function in people with A-T (48, 49).
• Pulse oximetry: Spot checks at routine surveillance clinical visits should be conducted using overnight saturation studies if there are any concerns regarding disordered breathing during sleep, worsening bronchiectasis, pulmonary aspiration (50), or suspicion of intestinal lung disease.
• Specialist radiology: DSBs can occur following exposure to ionizing radiation. For this reason, the need for x-rays should be carefully considered and be undertaken only if radiographic imaging is clinically needed to direct therapy. Thus, chest x-rays and computed tomography may occasionally be justifiable despite the increased radiosensitivity; however, the dose of radiation must be minimized under the supervision of an experienced pediatric radiologist. Having said this, the cardinal error of using such low-dose radiation that the images are uninterpretable must be avoided. Application of ionizing radiation-free imaging techniques for the assessment of the respiratory tract, including lung ultrasound (US) and lung magnetic resonance imaging (MRI) have a potential role in the assessment and monitoring of lung disease related to A-T. The lung US (51) can show changes such as multiple subpleural consolidations and B line artifacts related to the interstitial-alveolar syndrome and pleural effusion. Lung MRI (52) can detect mediastinum lymphadenopathy, bronchiectasis, peribronchial thickening, mucous plugging, and collapse/consolidation. In one study, total MRI score was higher in patients with respiratory symptoms, but abnormalities were present in all patients although only 53% had recurrent/chronic respiratory symptoms. MRI scores from patients with positive or negative sputum cultures were not significantly different. MRI has also been emerging as an imaging modality to assess ILD (53). MRI does hold promise as a modality that can be used on an annual basis to monitor lung health, detect any pulmonary changes, and monitor the effects of any treatment interventions. According to expert guidelines (5, 24, 25), imaging should be accompanied by regular lung function monitoring aimed at the early detection of lung disease progression and the need for therapeutic interventions. Ongoing studies (54) may inform us in the future about the age from which to start doing annual lung MRI scans, and the specific scanning sequences and functional techniques to monitor changes over time.
• It is assumed that the child will have access to all basic forms of support, including pediatric psychology, occupational therapy, social work, and play therapy, and all relevant professionals to ensure optimal access to services including school. The UK A-T service model is an innovative three-way partnership between national centers of expertise, local care teams, and patient organizations. The model provides a workable approach to address some of the logistic challenges that the patients and families encounter in this rare, complex, and life-limiting condition (55).

## Management of respiratory disease

This has been extensively reviewed in the ERS statement (25). The key points are as follows:

### Preventative measures to promote lung health

Preventative measures to promote lung health are essential and include avoidance of first- or second-hand cigarette smoke and electronic cigarette vapor exposure and full immunizations, including annual influenza and COVID-19 immunizations (see below). Inactivated vaccines are safe in ataxia-telangiectasia and are recommended as part of routine childhood vaccination programs, except for patients who are on immunoglobulin replacement therapy. Unless the diagnosis of A-T is made early, based on family history or by newborn screening, these children would have received live vaccination inadvertently, e.g., magnetic resonance imaging (MMR) or bacillus calmette-guérin (BCG). Clinical experience has been reassuring and adverse reactions to live vaccinations have not been described. However, as a general principle, live vaccinations, including the live attenuated influenza vaccine, should not be given to children or adolescents with A-T who are clinically severely immunocompromised.

Annual vaccination with an inactivated influenza vaccine is recommended for all patients, including those receiving immunoglobulin replacement therapy (56). However, experimental work shows that in mice, Atm is required to mount a proper memory response against the influenza virus by producing neutralizing virus-specific antibodies, T-cell memory, and resolution of inflammation following primary influenza virus infection. This implies that vaccination of children with A-T by itself may insufficiently protect against respiratory viral infections (57). Several studies have found a diminished response to pneumococcal vaccination. Expert guidelines recommend vaccination of all patients with ataxia-telangiectasia with a 13-valent pneumococcal conjugate vaccine followed by a 23-valent pneumococcal polysaccharide vaccine, repeated every 5 years (24, 25).

#### A-T and COVID-19

It is now very well-recognized that COVID-19 is associated with milder disease in children compared to adults. Even during the peak of the COVID-19 pandemic, studies (58–60) showed that pre-existing respiratory, immunological, or neurological disease in children (deemed clinically extremely vulnerable risk groups) did not appear to be a significant risk factor for severe COVID-19. Moeller et al. (60) reported that 284 out of 945 (30%) children required admission to hospital including 35 (4%) who were treated in PICU, only one of whom was a child with A-T. Out of the 247 A-T patients on the Iranian national registry, 36 patients (14.5%) had a confirmed COVID-19 infection. Of these, 35 were asymptomatic or showed mild symptoms. Only one child with both autosomal recessive A-T and X-linked recessive Toll-like receptor 7 deficiency required admission to a critical care unit (58).

##### COVID-19 immunization

None of the COVID-19 vaccines contain live virus. Children and young people (CYP) with A-T should receive a COVID-19 vaccine whether or not they previously had a COVID-19 infection, and whether or not they are receiving immunoglobulin replacement therapy. During the height of the pandemic, some specialist A-T centers suggested that a blood test for the level of the anti-COVID-19 antibody be performed 4–6 weeks after the second dose of vaccine to confirm protective antibody levels (61).

The recommendations in autumn 2024 (62) in the UK were that a booster dose should be given to individuals aged 6 months and over in a clinical risk group which includes anyone with chronic respiratory, neurological disease, or immunosuppression. Hence all individuals with A-T should be offered the vaccine.

##### Infection

Routine microbiological surveillance should be undertaken and additional samples obtained at the time of change in symptoms from the stable baseline. A-T lung disease has been associated with both bacterial and viral infections, but usually not opportunistic pathogens (19, 21, 22, 63). *Haemophilus influenzae*, *Moraxella catarrhalis, Streptococcus pneumoniae, Staphylococcus aureus,* and occasionally *Pseudomonas* species are the main pathogens in culture-positive patients (25). There is evidence of dysbiosis of the upper airway in these patients and increased numbers of *S. pneumoniae* (64). Yeo et al. reported that nasal epithelial cells from individuals with A-T were more sensitive to the damaging effects of infection with *S. pneumoniae* than controls and that this was partially due to the production of H_2_O_2_ by *S. pneumoniae* (64). Patients with A-T with elevated IgM levels (as compared to those with normal IgM levels) are more prone to chronic infection of the lower respiratory tract with pathogenic gram-negative bacteria *P. aeruginosa,* fungi, and non-tuberculous mycobacteria (NTM) (16). In these patients, respiratory infections manifest at a very young age and they have a more severe phenotype, as they are more likely to have autoimmune disease, more likely to be receiving immunoglobulin replacement therapy, and show decreased survival compared to patients with A-T with normal IgM levels (10–17).

##### Respiratory viruses

In a prospective case-controlled study where symptom questionnaires and nasopharyngeal swabs for respiratory viruses were obtained monthly, CYP with A-T were more frequently symptomatic in comparison with controls whether or not a respiratory virus was detected on routine microbiological surveillance (65).

Antibiotic treatment should be considered under the following circumstances:

• Any increase in respiratory symptoms, especially chronic wet cough, irrespective of whether there are any abnormalities heard with the stethoscope. A culture of respiratory secretions should be performed and airway clearance techniques be reviewed. Blind treatment with oral antibiotics should be commenced, guided by previous cultures. If there are no previous helpful results, then blind treatment with co-amoxiclav or another antibiotic which covers *H. influenzae*, *M. catarrhalis, S. pneumoniae,* and *S. aureus* should be given. We recommend the use of high doses (formerly a “serious infection” dose) for 2–4 weeks, continuing until the child has returned to baseline for at least 7 days or when the family feels that the child is more or less returned to baseline. Antibiotics can be changed depending on the culture results.
• Any positive upper or lower airway culture, especially for bacteria commonly cultured in people with A-T (*H. influenzae*, *S. pneumoniae*, and *P. aeruginosa*) (22) should be treated with 2–4 weeks of an appropriate oral antibiotic as above, even if the child is asymptomatic. It is known that there is an increased susceptibility of airway epithelial cells to *S. pneumoniae* infection in ataxia-telangiectasia (64). In addition, patients with primary antibody deficiency have a significant respiratory symptom burden associated with increased viral infection and a more frequent presence of *H. influenzae* and *S. pneumoniae* in the upper respiratory swabs despite immunoglobulin replacement and prophylactic antibiotic use (66). However, it has been shown that positive upper airway cultures bear no relation to lower airway cultures, inflammation, or structural lung damage in the lungs at the time that the cultures are taken or 1 year later in children with CF (67). Although the relationship between upper and lower airway cultures has not been studied in A-T, and despite the weak relationship in other diseases, our (non-evidence-based) practice would be to treat a positive upper airway culture. Whether a greater use of induced sputum, as in CF (68), would improve outcomes is a matter for future research.
• If the response to oral antibiotics is inadequate, or the child is very unwell, then admission for intravenous antibiotics and intensive airway clearance is mandated. The choice of intravenous antibiotics will depend on cultures or a best guess. The isolation of *P. aeruginosa* should be treated with a CF-protocol eradication regime (69), for example, 3 weeks of oral ciprofloxacin and 3 months of nebulized colistin.
• In a child with a chronic productive cough despite trials of antibiotics, especially if they are culture-negative, consideration should be given to an induced sputum sample or fiberoptic bronchoscopy (FOB) and bronchoalveolar lavage (BAL) to obtain material for culture. Opportunistic infections are rare in A-T, but if suspected, early FOB and BAL are mandated.
• Children with A-T are also prone to sinusitis and otitis media, and these should also be treated with prolonged courses of antibiotics. In those patients with frequent exacerbations or who are refractory to treatment, specialist referral for sinonasal MRI, nasoendoscopy, and sinoscopy will help guide and optimize treatment. Sinus CT should be avoided if possible, because of the radiosensitivity which is intrinsic to A-T.

Long-term maintenance antibiotic treatment is considered on an individual basis when the burden of respiratory infections is high, but microbiological resistance and side-effects should be always borne in mind. When indicated, azithromycin is commonly used, an extrapolation from other primary immunodeficiency disorders (70). Azithromycin may already have been commenced by the treating immunologist.

#### Immunoglobulin replacement

Immunoglobulin replacement therapy is indicated in all patients with a severe immunoglobulin-G deficiency (serum immunoglobulin-G < 4 g/L) and in those with a high IgM phenotype (19, 71); it should also be considered for patients with milder humoral immune defects, recurrent infections, or low specific antibody responses despite booster immunization (56). However, published studies report a wide variation in practice with between 13% and 60% of patients with A-T receiving replacement immunoglobulin therapy (25).

##### Respiratory physiotherapy

Measures to improve airway clearance and prevent atelectasis are likely to be beneficial in patients with A-T, especially in those with more advanced neurological decline, as they have both impaired secondary mucociliary and cough clearance (25). Although not specifically studied in A-T, it is well known that pollutants, common bacterial pathogens, and respiratory viruses can affect normal ciliary function. A number of mechanisms may underlie this, including the production of specific ciliotoxins, which impair ciliary motion and coordination, disrupt the microtubule function of ciliated columnar cells, and change the viscosity of airway mucus. Furthermore, inflammatory cytokines in the presence of infection can also negatively modulate cilia function (72). As in other neuromuscular disease disorders, treatment regimens may be intensified during episodes of acute illness. Physiotherapy advice regarding which techniques to adopt to optimize airway clearance before irreversible structural lung damage occurs is desirable, although there is no firm evidence base to give specific advice for patients with A-T. There has been only one longitudinal study (73) that assessed spirometry, subjective sensation of dyspnea, maximal inspiratory pressure (MIP), maximal expiratory pressure (MEP), and quality of life (QoL) before and after a 24-week inspiratory muscle training program in 11 patients with A-T and nine healthy volunteers. The results showed that inspiratory muscle training was effective in improving ventilatory pattern, lung volume, respiratory muscle strength, and the health and vitality domains of quality of life in patients with A-T. Inspiratory muscle training was an effective adjunct therapy to drug treatment for patients with A-T. A recent scoping review on multidisciplinary team (MDT) care in five non-research sources of evidence found that common recommendations included improving pulmonary function and reducing aspiration within a multidisciplinary approach (24, 25, 74–76) with an emphasis on regular activities and breathing exercises for optimizing respiratory function. Hence, it would be considered good practice to advise some form of regular exercise, even in younger children who are still mobile and not yet on a downward trajectory of pulmonary and neurological decline. Physical therapy and exercise should not be used to the point of fatigue. Children and adolescents with A-T often complain of progressive daily fatigue which can be exacerbated by physical activities. The etiology of the fatigue is not completely understood but its presence and severity may be associated with progressive neurological deterioration. Other causes of fatigue can include disruption or fragmentation of nighttime sleep, poor nutrition, depression, or other medical causes (5). A range of interventions (and suggestions to reduce the potential for tripping and falling and avoid injuries) that positively impact ataxia-related impairments, activity, or participation levels, together with QoL measures, have been very well summarized in a recent review (77).

There are no studies into the efficacy of nebulized hypertonic saline, rhDNase, or inhaled mannitol on the mobilization of mucus in A-T. The prescription of nebulized saline in CYP with neurodisabilities with a median age of 11 years (none with A-T) was associated with improved respiratory outcomes in a retrospective study (a decrease in the number of hospitalizations per year and a decrease in total courses of antibiotics for chest infections per year) and was favorably received by the patients and their caregivers (78). Most patients in this study were administered combinations of treatment with different concentrations of saline including isotonic or hypertonic saline (3%–7%) and so a dose effect could not be demonstrated. If tolerated, we would initiate treatments with 7% hypertonic saline.

##### Reversible airflow obstruction

Documentation of an acute response to bronchodilators is not uncommon, and, of course, A-T does not protect a child from also developing asthma. There is no evidence that prescribing inhaled corticosteroids (ICS) to children with A-T and reversible airflow obstruction but no risk factors for asthma, is beneficial. It is suggested that if a trial of ICS is contemplated, it should be for a finite period with definite end-points before the child is committed to long-term therapy. As with any other child, evidence for eosinophilic airway disease should be sought before trialing ICS (Table 3). It should be noted that there is increasing evidence that ICS may increase the risk of airway infection in other contexts, and hence there is a reason for caution in long-term use.

**Table 3:** Evidence supporting coincident eosinophilic airway inflammation in A-T.

Technique	Comment
Raised peripheral blood eosinophil count	Non-specific—can be elevated in other non-asthma atopic disease and parasite infections.
Exhaled nitric oxide	Can be collected during tidal breathing even in those who are neurologically impaired Can also be elevated in atopy without asthma.
Atopic sensitization (skin prick tests, specific IgE)	Eosinophilic airway inflammation rare in non-atopic children.
Eosinophilia of induced or spontaneously expectorated sputum	The gold standard but induced sputum not a routine test.

##### Disordered breathing during sleep

Cardiorespiratory polysomnography (CRPS) should be considered if there is a clinical suspicion of sleep-related breathing abnormalities, rapid or progressive decline in lung function, or if developing scoliosis. As with any child with scoliosis, rapid progression while going through puberty may be seen. If significant sleep-related disordered breathing is confirmed on CRPS, the decision to undertake non-invasive ventilation (NIV) should be made on an individualized basis, based on the benefit outweighing the risk (for example, nocturnal aspiration) and the burden of the treatment.

##### Interstitial lung disease

Interstitial lung disease in patients with A-T is rare; a definitive diagnosis of ILD was made in 25 out of 97 patients with A-T who had either chronic respiratory symptoms or pulmonary disease listed as a cause of death (out of a total of 437 patients with A-T) (79). The onset of ILD usually occurs in adolescence and is characterized by non-specific symptoms but should be suspected if the child has a persistent *dry* cough, breathlessness, persistent and especially fine crackles in the absence of respiratory infection, or oxygen desaturation. ILD should be also suspected when pulmonary disease does not respond to antibiotic or bronchodilator treatment (79). A bronchoalveolar lavage and lung biopsy may be helpful in confirming microbiological causes of infections and ILD, respectively (25).

The mortality rate related to ILD in patients with ataxia-telangiectasia is approximately 80%. Aspiration and immunodeficiency do not play a role in the development of ILD, whereas chemotherapeutic drugs such as bleomycin may cause a type of ILD with pulmonary fibrosis dominating (80–82). The histological findings in patients with ataxia-telangiectasia and ILD are unique and do not fit the current classification systems for ILD but could fit in the proposed other known or well-characterized non-genetic or as yet unknown category (83). One should keep in mind that pulmonary involvement of lymphoma may mimic ILD (84).

The evidence base for an investigation pathway is even more scarce but since the treatment is a high dose of oral corticosteroids, most would advocate obtaining a tissue diagnosis before treatment. This would involve a low radiation dose CT scan (ensuring sufficient radiation to acquire adequate images). Other techniques include photon-counting CT which can reduce radiation exposure and reconstruct images at a higher resolution (85), but more evidence its utility in A-T is needed. Imaging should be followed up with video-assisted thoracoscopic surgery (VATS) or an open lung biopsy. Such evidence as exists suggests that early treatment with oral corticosteroids is most beneficial (79) so diagnosis and treatment should be aggressively pursued at an early stage.

#### Other non-infective respiratory manifestations

Bronchiolitis obliterans has also been described in ataxia-telangiectasia (86). Pleural neoplasms are extremely rare in children, even in patients with A-T, and hence delayed diagnosis of conditions such as malignant pleural mesothelioma can occur in these patients (87). Parenchymal lung involvement is not uncommon in Hodgkin's disease, one of the well-described hematological malignancies in A-T; however, pulmonary infiltration with cavitation in the lung parenchyma is quite unusual and can be associated with a fatal outcome (84).

##### Planning for surgical procedures

The term ataxia-telangiectasia cannot be found in a British Thoracic Society comprehensive document (88) on respiratory management of children with neuromuscular weakness, highlighting the lack of evidence in managing this rare disorder. Surgery (for gastrostomy insertion or scoliosis) in children with A-T should take place in units with experienced pediatric surgeons, anesthetists, and physiotherapists, and where there are facilities for pediatric intensive care and NIV. These children should be assessed by a multidisciplinary team prior to any intervention. Anesthesia carries risks for these patients comparable to other medically complex patients. Precautions include a full evaluation of the following potentially impaired bodily systems prior to anesthesia:

• Neurological—in particular assessing cerebellar and bulbar function, minimizing the risk of aspiration during the induction of anesthesia.
• Respiratory—seeking and treating infection, undertaking a sleep study, and considering whether the child might need NIV postoperatively, and if so, familiarize themselves with it preoperatively.
• Cardiovascular evaluation to be aware of the potential effects of increased pulmonary vascular resistance, which may be a complication of chronic lung disease.
• Hematological—looking for malignancies resulting in pancytopenia and necessitating transfusion of blood and platelets preoperatively.
• Metabolic—diabetes has been found to be present in 18% of patients with A-T after puberty (89) and would require careful perioperative management.
• Optimize nutrition.

Early reports suggested high perioperative morbidity in A-T (75), while the results of the largest published series are more favorable, reporting that general anesthesia, airway manipulation, and perioperative mechanical ventilation may be tolerated with only minor postoperative anesthetic concerns (90) even when anesthesia was required for the surgical management of recurrent infective pleural effusions (91). Effective airway clearance techniques, and early or even immediate use of NIV following extubation should be anticipated and planned. The outcomes of invasive ventilation are less good with case reports of adverse outcomes (92). Given the likelihood of amplified damage from oxidative stress due to the abnormal DNA damage response (4), the recent suggestion of a conservative oxygenation saturation target (SpO_2_ 88%–92%) could be very relevant if a child with A-T was admitted and invasively ventilated (93).

#### Growth and nutrition

Undernutrition adversely affects lung health. Poor nutritional status and decreased pulmonary function have been shown to be linked in other diseases, including CF. Worsening nutritional status increases infection-related morbidity and mortality. Malnutrition is of particular concern in children since it adversely affects the normal accrual of height and weight and may impact lung development.

Several studies have shown that patients with A-T exhibit high rates of malnutrition, short stature, and reduced lean body mass (94–101). Worsening nutritional status over time in CYP with A-T can be progressive in a third to half of patients (99, 101).

Early gastrostomy insertion stabilizes nutrition decline, improves growth, minimizes the risk of aspiration, is better tolerated, and improves the quality of life of CYP with A-T and that of their caregivers (99, 102).

#### Swallowing/aspiration assessment

Aspiration occurs in many patients, especially in those who are older and malnourished (104).

**Table 4:** A penetration–aspiration scale for assessment of swallowing and aspiration (103).

Oral-motor clinical assessment	Lip closure
	Tongue movement
	Palatal elevation
	Gag reflex
	Voice quality
	Speech motor control
**Clinical swallowing assessment** using a multidimensional tool designed to measure both the supervision level required and the diet level	1. Individuals are not able to swallow safely by mouth. All nutrition and hydration is received through non-oral means (e.g., nasogastric tube).
	2. The individual is not able to swallow safely by mouth for nutrition and hydration but may swallow some consistently with consistent maximal cues in therapy only. An alternative method of feeding is required.
	3. Alternative method of feeding required as the individual receives less than 50% of their nutrition and hydration by mouth and/or swallowing is safe with consistent use of moderate cues to use compensatory strategies and/or requires maximum diet restriction.
	4. Swallowing is safe but usually requires moderate cues to use compensatory strategies and/or the individual has moderate diet restriction and/or still requires tube feed­ing and/or oral supplements.
	5. Swallowing is safe with minimal diet restriction and/or occasionally requires minimal cueing to use compensatory strategies. May occasionally self-cue. All their nutrition and hydration needs are met by mouth at mealtime.
	6. Swallowing is safe and the individual eats and drinks independently and may rarely require minimal cueing. Usually, the individual self-cues when a difficulty occurs. May need to avoid specific food items (e.g., popcorn and nuts) or requires additional time (due to dysphagia).
	7. The individual’s ability to eat independently is not limited by their swallowing function. Swallowing would be safe and efficient for all consistencies. Compensatory strategies are used effectively when needed.
Penetration/aspiration assessment (103)	1. Material does not enter the airway.
	2. Material enters the airway, remains above the vocal folds, and is ejected from the airway.
	3. Material enters the airway, remains above the vocal folds, and is not ejected from the airway.
	4. Material enters the airway, touches the vocal folds, and is ejected from the airway.
	5. Material enters the airway, touches the vocal folds, and is not ejected from the airway.
	6. Material enters the airway, passes below the vocal folds, and is ejected into the larynx or out of the airway.
	7. Material enters the airway, passes below the vocal folds, and is not ejected from the trachea despite effort.
	8. Material enters the airway, passes below the vocal folds, and no effort is made to eject it.

Early detection and treatment of swallowing dysfunction appear to decrease morbidity associated with dysphagia and enable early oral rehabilitation (i.e., compensatory strategies and diet modification), which in turn could decrease the risk of aspiration pneumonia and improve the quality of life of affected individuals. The standard means of swallowing assessment with a videofluoroscopic swallow study (VFSS) are limited by concerns about radiation exposure and a cumulative dose of radiation exposure that may result from multiple studies, especially in a condition such as A-T with increased radiation sensitivity (104).

Swallowing and aspiration-related problems become more prevalent as the neurological difficulties progress. Symptoms of unsafe swallowing include coughing and choking during meals, incoordination between breathing and swallowing events, absence of the cough reflex, change in voice quality (hoarse, wet/crackly, or weak voice), and difficulty in maintaining weight or loss of weight. A review by an experienced speech and language therapist should include an oral-motor assessment and a clinical swallowing assessment, preferably using a scale that describes penetration and aspiration (Table 4) (103). Low-dose, modified VFSS may be used in exceptional cases (104, 105); however, a clinical assessment that avoids the use of ionizing radiation would be preferable. The use of cervical auscultation of swallowing and respiratory sounds and/or vibrations has been shown to have a high sensitivity and specificity in the detection of oropharyngeal aspiration (106). Other methods that do not require the use of ionizing radiation include fiberoptic endoscopic evaluation of swallowing (FEES) (106) or videostroboscopy (107) to provide improved dynamic visualization of the glottis. However, both these methods require special expertise and equipment and data in A-T are lacking.

More advanced methods of assessment such as using a respirodeglutometer (RDG DFI Enterprises, Inc., Morrisville, NY, USA) provide valuable insight into the physiological basis of respiration–swallowing coordination. However, this is not used in routine clinical care, and, at present, is primarily a research technique. These recordings provide non-invasive measures of respiratory airflow during swallowing. Assessment may allow initiation of treatment before the development of sequelae in persons at increased risk for the development of aspiration. An expiration–swallow–expiration (E–Sw–E) pattern of peri-deglutitive airflow is considered the safest pattern of coupling for swallowing; inspiration-swallow-inspiration (I-Sw-I), the least safe pattern. Regardless of age, E–Sw–E was the most common pattern for both patients with ataxia-telangiectasia and healthy participants. In a small study in a specialized A-T center, patients with A-T were shown to have proportionately fewer E–Sw–E airflow patterns than their age-matched healthy controls; they generated more frequent inspiratory airflow patterns either before deglutition, after deglutition, or both. Furthermore, the least safe pattern, I–Sw–I, occurred significantly more often in older patients with A-T than in the controls (108).

Early expert input from a speech and language therapist to implement changes in feeding routine such as elimination of thin liquids, pacing to slow the rate of liquid intake, and having easily chewable food, among others, is valuable. Advice about body posture to maintain an upright stable position to avoid shortening of the neck during eating and drinking is crucial (25).

#### What is on the horizon?

It is beyond the remit of this review to discuss future research in detail. We will very briefly highlight some of the current ongoing research that may have disease-modifying potential in the future.

• Genetic modification: A study on the use of personalized antisense oligonucleotides (ASOs) in a 3-year-old girl with A-T is still ongoing and may become a promising disease-modifying treatment (109). The large size of the ATM protein has so far eluded viral vector-based gene therapy, which has shown some promise in spinal muscular atrophy (110) and Tay–Sachs disease (111).
• Stem cell/bone marrow transplant: Pre-emptive allogeneic hematopoietic stem cell transplant (alloHSCT) led to the correction of immunodeficiency in a 4-year-old boy with A-T. An acceptable risk of transplant-related mortality was reported in this case report (112). However, the risks of life-threatening events associated with the drugs and radiation used for pre-transplant preparative regimens are not insignificant (113)
• A recent review (114) has summarized some other treatment approaches that target oxidative stress and inflammation. Experimental work has shown the potential for triheptanoin, a synthetic medium-chain triglyceride (115), to correct metabolic stress in A-T cells.

## Summary

We have provided an overview of respiratory morbidity in this ultrarare disease. There are multiple contributors to respiratory disease in A-T and respiratory disease is the leading cause of death in patients with A-T along with cancer. Regular proactive measures are recommended to detect and manage lung disease in patients with A-T in an attempt to modify the impact of this devastating condition and an unpublished work (personal communication) suggests that more young people with A-T are being transitioned to adult A-T clinics with this proactive approach.
